# Transcriptome profiling of mouse colonic eosinophils reveals a key role for eosinophils in the induction of s100a8 and s100a9 in mucosal healing

**DOI:** 10.1038/s41598-017-07738-z

**Published:** 2017-08-02

**Authors:** Hadar Reichman, Italy Moshkovits, Michal Itan, Metsada Pasmanik-Chor, Thomas Vogl, Johannes Roth, Ariel Munitz

**Affiliations:** 1 Department of Clinical Microbiology and Immunology, The Sackler School of Medicine, Tel-Aviv University, Ramat Aviv, 69978 Israel; 2 Bioinformatics Unit, George S. Wise Faculty of Life Sciences, Tel Aviv University, Tel Aviv, 64239 Israel; 3 Institute of Immunology, University of Münster, Münster, Germany

## Abstract

Eosinophils are bone marrow-derived cells that have been largely implicated in Th2-associated diseases. Recent data highlights a key role for eosinophils in mucosal innate immune responses especially in the gastrointestinal (GI) tract, which is one of the largest eosinophil reservoirs in the body. Although eosinophils express and synthesize a plethora of proteins that can mediate their effector activities, the transcriptome signature of eosinophils in mucosal inflammation and subsequent repair has been considerably overlooked. We demonstrate that eosinophils are recruited to the colon in acute inflammatory stages where they promote intestinal inflammation and remain in substantial numbers throughout the mucosal healing process. Microarray analysis of primary colonic eosinophils that were sorted at distinct stages of mucosal inflammation and repair revealed dynamic regulation of colonic eosinophil mRNA expression. The clinically relevant genes s100a8 and s100a9 were strikingly increased in colonic eosinophils (up to 550-fold and 80-fold, respectively). Furthermore, local and systemic expression of s100a8 and s100a9 were nearly diminished in eosinophil-deficient ΔdblGATA mice, and were re-constituted upon adoptive transfer of eosinophils. Taken together, these data may provide new insight into the involvement of eosinophils in colonic inflammation and repair, which may have diagnostic and therapeutic implications.

## Introduction

Eosinophils are bone marrow (BM)–derived granulocytes that differentiate primarily under the regulation of interleukin 5 (IL-5)^1^. At baseline, eosinophils mainly reside (at substantial levels) in tissues such as the gastrointestinal (GI) tract, spleen, adipose tissue, lymph nodes and thymus^2^. Eosinophils have been traditionally studied in the context of allergic inflammatory settings since their accumulation is a hallmark disease feature. Yet, the GI tract is the largest eosinophil reservoir in the body and recent data emphasize key roles for eosinophils in GI homeostasis and multiple inflammatory conditions including those associated with inflammatory bowel disease (IBD) [e.g. ulcerative colitis (UC) and Crohn’s disease (CD)]^3^.

Previous studies investigating eosinophils in context of colonic inflammation were mainly focused on assessing triggers that recruit eosinophils and their overall function. For example, following dextran sulphate sodium (DSS) treatment, eosinophils are actively recruited to the inflamed colon by the CCR3:eotaxin pathway^4^, which is mediated by activation of RelA/p65 in myeloid cells^5,6^. Eosinophilia was also reported in SAMP1/Yit mice (which develop ileitis resembling CD)^7^, in oxazolone-induced colitis and in *Il10*^−/−^ mice, which spontaneously develop colitis^8^. Mechanistically, targeted ablation of eosinophils in mice using two independent mouse models (namely the *Phil* and ΔdblGATA mice)^4,9,10^ resulted in decreased DSS-induced disease progression^4,11,12^. Furthermore, mice deficient in eosinophil-specific granule proteins such as eosinophil peroxidase and eosinophil major basic protein displayed attenuated disease phenotype in colitis^8,9^. In fact, accumulation of eosinophils in the inflamed colon has been associated with the levels of calprotectin (heterodimer of s1000a8 and s100a9)^6^, which serves as a clinical diagnostic marker for active colonic inflammation. Intriguingly, a recent study demonstrated a protective effect for eosinophils in acute mouse colitis, via production of anti-inflammatory lipid mediators^13^. Collectively, these findings reflect the multifaceted role of eosinophils during colonic inflammation.

Over the past years, the subject of intestinal healing has drawn considerable attention and has been recommended as a new treatment goal in IBD. Mechanistically, mucosal healing is defined as suppression of the inflammatory process and the restoration of the intestinal barrier ultimately leading to disease remission. Importantly, although eosinophils can mediate tissue damage they are also capable of tissue healing and repair^14^. Eosinophils are a chief source for fibrotic factors including Relm-α^15–17^ and TGF-β, which is linked with epithelial growth^18,19^, fibrosis, and tissue remodeling in the lungs and esophagus of asthmatic and eosinophilic esophagitis patients^20–23^. Furthermore, eosinophils produce VEGF and are capable of promoting endothelial cell sprouting^24,25^. Finally, eosinophils have been recently associated with liver and muscle regeneration by regulating the activities of macrophages by secretion of IL-4^26,27^.

Although the role of eosinophils has been assessed experimentally in the acute inflammatory stages of colitis, their roles in mucosal healing have been largely unexplored. Furthermore, the phenotypic landscape of eosinophils in distinct stages of colonic inflammation (i.e. inflammatory stage vs. the repair stage) remain to be characterized.

In the present study, we demonstrate that eosinophils are recruited to the colon during acute colonic inflammation and are present in substantial numbers also throughout intestinal healing and repair stage. Global transcriptome profiling of eosinophils from distinct stages of inflammation and repair revealed a unique phenotype for eosinophils during colonic inflammation, which is distinct from that of eosinophils in the repair stage. Furthermore, we show that eosinophils highly upregulate their expression of s100a8 and s100a9 and that the expression of these proteins in colonic inflammation and repair is largely eosinophil-dependent. These studies provide new insight into eosinophil-mediated effector pathways in colonic inflammation and repair, which may provide new targets for prevention and treatment of colitis and perhaps colorectal cancer.

## Results

### Eosinophils are present in the colon during mucosal healing

**Figure 1:**
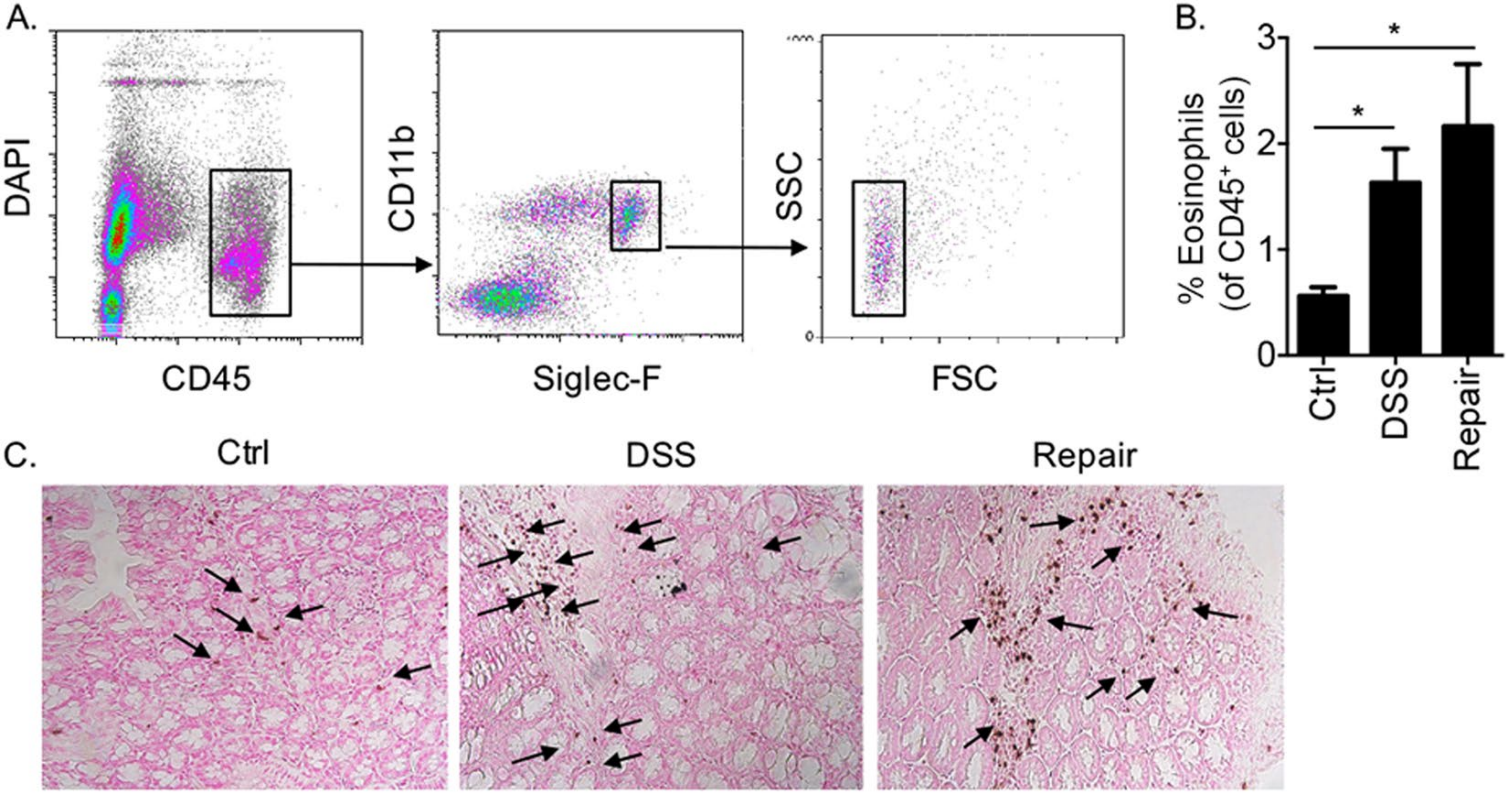
Eosinophils are present in the colon during mucosal healing. Gating strategy for identification of colonic eosinophils in the lamina propria following enzymatic digestion (**A**). The percent of eosinophils during colonic inflammation (DSS) and Repair (**B**) as well as representative photomicrographs of H&E-stained slides from control (Ctrl) mice and mice in the inflammation and repair stages (**C**). Data in (**A**) and (**C**) are representative of at least n = 3; Data in **B** are from at least n = 10 mice, *p < 0.05.

**Figure 2:**
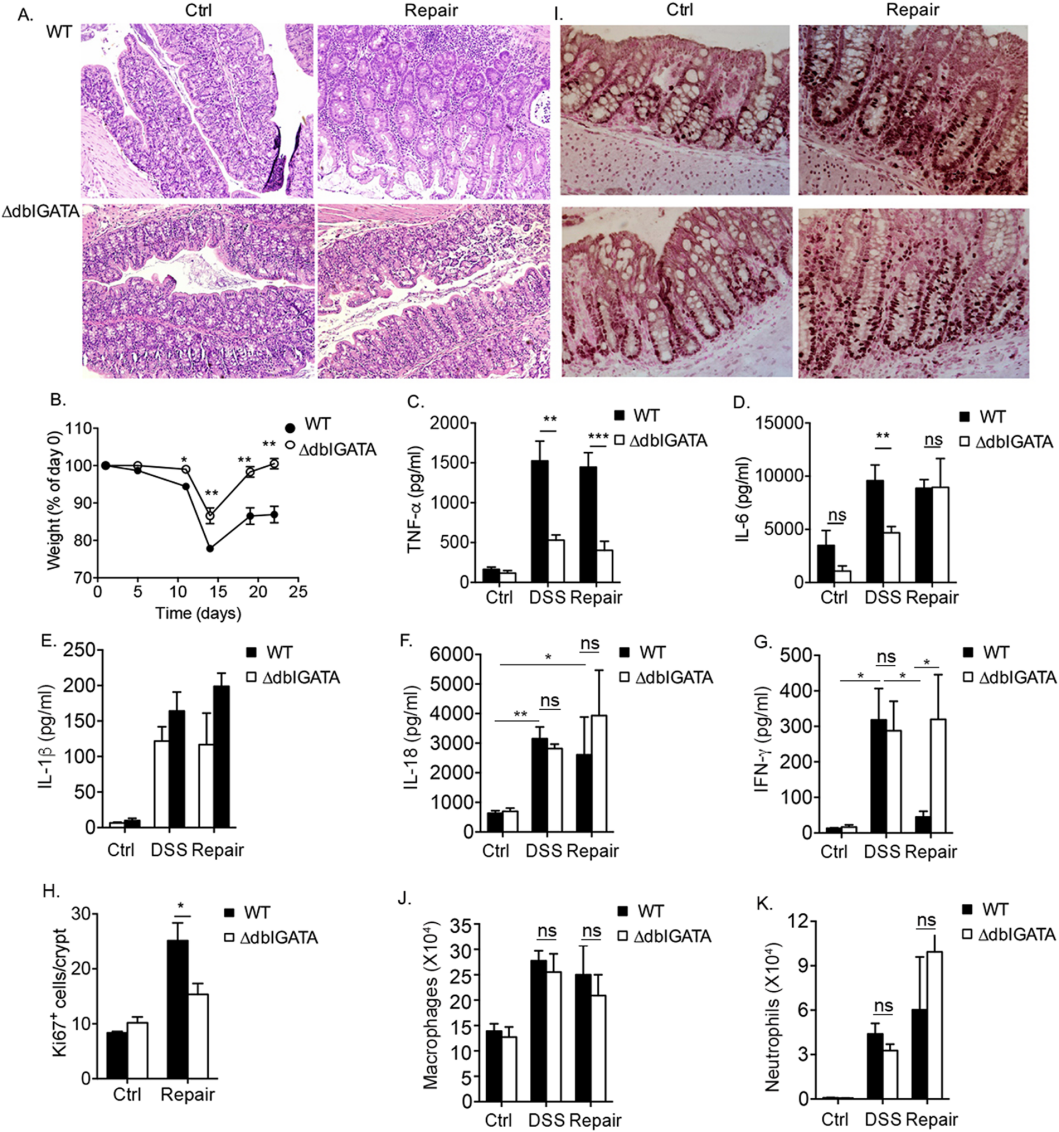
Accelerated mucosal healing following DSS treatment in the absence of eosinophils. Representative H&E-stained (**A**) and Ki-67-stained slides (I) from control (Ctrl) mice and mice during the repair stage. The weight of wild type (WT) and eosinophil deficient mice (ΔdblGATA mice) was determined at the indicated time points during the experimental regimen (**B**). The expression of TNF-α (**C**), IL-6 (**D**), IL-1β (**E**), IL-18 (**F**) IFN-γ (**G**) and quantitative analysis of Ki-67^+^ cells (**H**) is shown. Analysis of macrophage (**J**) and neutrophil (**K**) infiltration in the colons of wild type and ∆dblGATA mice under baseline conditions (Ctrl), following DSS-treatment and during the repair phase. Data in (**I**) and (**E**) are representative of at least n = 3, other data are from at least n = 3 experiments, ***p < 0.001; **p < 0.01, *p < 0.05, ns- non-significant.

Flow cytometric analysis of colonic DAPI^−^/CD45^+^/Siglec-F^+^/CD11b^+^/SSC^high^ cells, defined as eosinophils^28^ (Fig. 1A) revealed marked elevation of eosinophils upon DSS treatment, which was further elevated fourteen days after cessation of DSS treatment (Fig. 2B). Accumulation of eosinophils in the lamina propria of the colon during inflammation and repair was further validated by anti-eosinophil major basic protein immunohistochemistry (Fig. 1C).

### Accelerated mucosal healing following DSS treatment in the absence of eosinophils

Consistent with previous studies^4,5^, DSS-treated ΔdblGATA mice displayed significantly decreased mucosal pathology as could be seen by the attenuated inflammatory infiltrate, crypt loss, and epithelial erosion which were evident in the acute inflammatory stage and later during mucosal repair in wild type mice (Fig. 2A). Withdrawal of DSS from the drinking water of ΔdblGATA mice resulted in accelerated repair in comparison with wild type mice (Fig. 2A). In fact, ΔdblGATA mice fully regained their weight (Fig. 2B) and epithelial cell integrity was nearly completely reestablished with minimal cellular infiltrate (Fig. 2A). Since eosinophils were reported to display pro-inflammatory properties in DSS-induced colitis^4,9^, we next aimed to determine the role of eosinophils in regulating the expression of various pro-inflammatory cytokines that have been attributed key roles in colonic inflammation^29^. DSS-treated wild type mice displayed increased levels of TNF-α, IL-6, IL-1β, IL-18, which remained elevated also during the repair period (Fig. 2C–G). DSS-treated ΔdblGATA mice displayed decreased expression of the pro inflammatory cytokines TNF-α and IL-6 in colonic “punch” biopsies (Fig. 2C,D). Nonetheless, while the expression of TNF-α in punch biopsies of ΔdblGATA mice was significantly lower than those observed in wild type mice throughout the entire experimental regime (i.e. during acute inflammation and repair, Fig. 2C), IL-6 levels were lower only in the inflammatory stage (Fig. 2D). ΔdblGATA mice displayed no difference in the levels of IL-1β and IL-18 during the acute inflammatory response and following mucosal repair in comparison with wild type mice (Fig. 2E,F). Interestingly, IFN-γ displayed a different expression pattern when compared with IL-6, TNF-α, IL-1β and IL-18. In wild type mice, IFN-γ expression was increased during the inflammatory stage, and nearly completely decreased during the repair period (Fig. 2G). In contrast to wild type mice, ΔdblGATA mice exhibited elevated IFN-γ expression during the repair phase. Quantitation of Ki-67^+^ epithelial cells revealed that ΔdblGATA mice showed decreased epithelial cell proliferation during the repair stage when compared to wild type mice (Fig. 2H,I).

Next, we aimed to define whether eosinophils regulate the infiltration of additional inflammatory cells such as neutrophils and macrophages that may also affect colonic inflammation and repair processes^30–32^. To this end, single cell suspensions were obtained from the colons wild type and ΔdblGATA mice at baseline, following DSS-treatment and during the repair stage. No differences were observed between wild type and ΔdblGATA mice in the levels of infiltrating neutrophils (defined as: CD45^+^/CD11b^+^/Ly6c^−^/Ly6G^+^/MHC-II^−^/SSC^int^) and macrophages (defines as: CD45^+^/CD11b^+^/Ly6c^−^/Ly6G^−^/MHC-II^+^/SSC^hi^/FSC^hi^)^28^.

Collectively, these data suggested that eosinophils may directly regulate distinct features of inflammation and subsequent repair in the colon in part by regulation of cytokine production in the colon.

### Microarray analysis of eosinophils during inflammation and repair

**Figure 3:**
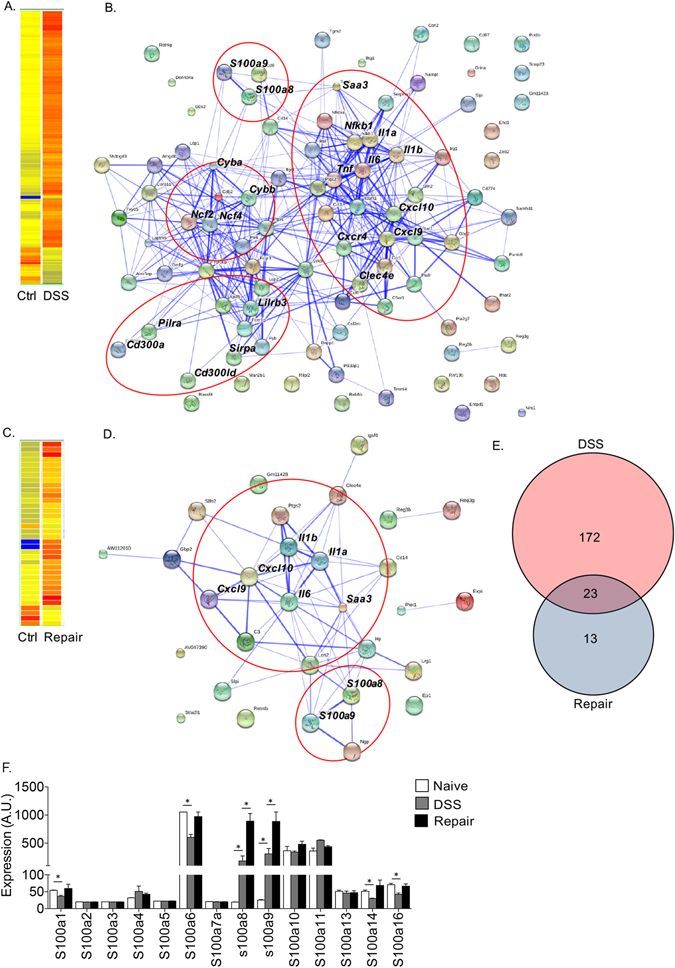
Microarray analysis of eosinophils during inflammation and repair. Colonic eosinophils were isolated from control (Ctrl) mice, and mice during the inflammatory (DSS) and healing (Repair) stages. Heat map (**A,C**) and STRING (**B,D**) analyses of genes that were significantly up-regulated (p < 0.05 and fold-change > 2, respectively) in eosinophils during the inflammatory stage (**A,B**) and tissue-repair stages (**C,D**). In (**E**), Venn diagram of the altered genes is shown. Relative expression of all s100a transcripts, which were identified in eosinophils following microarray analysis. The relative expression (as obtained from the microarray analysis) of transcripts encoding different s100a family members in eosinophils is shown (**F**); In (**F**), data are from n = 2 (each group consisting of eosinophils that were sorted from 5-10 mice); A.U – arbitrary units, *p < 0.05.

To gain insight into the phenotypic landscape of colonic eosinophils during inflammation and repair, primary colonic eosinophils were sorted after five days of DSS treatment or fourteen days after cessation of DSS treatment and subjected to microarray analysis. In comparison with eosinophils from control mice, eosinophils during the inflammatory stage displayed multiple differentially expressed transcripts. Among these 169 were upregulated and 27 downregulated (Fig. 3A and Supplemental Table 1). Un-biased STRING analysis, which identifies known and predicted protein interactions^33,34^, revealed that the transcriptome signature of eosinophils from the inflammatory stage was largely divided into four distinct clusters (Fig. 3B and Table 1). Cluster 1 comprised of transcripts encoding proinflammatory cytokines and chemokines; Cluster 2 included transcripts encoding the hallmark alarmins s100a8 and s100a9^35^; Cluster 3 consisted of various myeloid-associated immunoreceptors; Cluster 4 contained transcripts associated with NADPH oxidase activity.

**Table 1:** Representative eosinophil genes associated with acute inflammation and repair.

Cluster #	Cluster Title		Fold Change	
			DSS	Repair
1	Cytokines and Chemokines	*Cxcl9*	4.37	3.37
		*Cxcl10*	3.36	2.11
		*Il1a*	3.45	2.26
		*Il1b*	3.04	2.39
		*Il6*	2.66	2.24
		*Nfkb1*	2.09	1.19
		*Tnf*	2.52	1.71
		*Il1r2*	2.05	1.13
		*Il2rg*	2.56	1.25
		*Ccl3*	2.6	1.42
		*Ccl4*	2.34	1.66
		*Ccl6*	2.33	0.93
		*Ccr1*	2.4	1.23
		*Ccr3*	3.77	1.05
2	Alarmins	*s100a8*	8.21	45.3
		*s100a9*	11.6	34.3
3	Immunoreceptors	*Cd300a*	2.2	1.03
		*Cd300ld*	2.06	0.96
		*Pilra*	2.75	1.35
		*Sirpa*	2.5	0.95
		*Lilrb3*	2.21	1.30
4	NADPH oxidase activity	*Ncf2*	2.44	0.98
		*Ncf4*	2.39	1.16
		*Cyba*	2.9	1.66
		*Cybb*	4.2	1.41

In contrast to eosinophils from the inflammatory stage, eosinophils during the repair period displayed marked alterations only in 36 transcripts among them 31 were upregulated and 5 downregulated (Fig. 3C). Subsequent STRING analysis revealed that the transcriptome signature of eosinophils from the repair stage consisted of two major clusters (Fig. 3D and Table 1). Similar to eosinophils from the inflammatory stage, the first cluster comprised of transcripts encoding for proinflammatory cytokines and chemokines. Nonetheless, majority of these upregulated genes was relatively silenced at the repair stage. For example, the expression of *Cxcl9* and *Cxcl10*, which were increased in eosinophils in the inflammatory stage by 4.37- and 3.36-fold, decreased in in repair stage by ~30% to 3.37- and 2.11-fold. Furthermore, the expression of *Tnfa*, *CCl4* and *CCl3*, which were significantly increased (2.52-, 2.6- and 2.34-fold, respectively) in the inflammatory stage, decreased and their expression was not statistically different than their expression in eosinophils from control mice (Table 1, gray transcripts). The second cluster of eosinophils from the repair stage contained the alarmins s100a8 and s100a9. Remarkably, the expression of s100a8 and s100a9 dramatically increased during the repair stage even in comparison to eosinophils from the inflammatory stage. In fact, s100a8 and s100a9 displayed a further 5.5- and 2.9-fold (from 8.21 to 45.3 and 11.6 to 34.3), increase, respectively (Table 1). Analysis of the microarray data regarding the expression pattern of additional s100a family members revealed that although eosinophils express transcripts for various s100a family members, s100a8 and s100a9 were the only s100a family members displaying such a unique upregulation (Fig. 3F).

Further comparison of the differentially expressed transcripts revealed that the signature of eosinophils in the inflammatory stage was distinct since 88% of the identified transcripts (172 out of 195) were specific (Fig. 3E, Supplementary Table 1) whereas only 27% (13 out of 36) were unique to eosinophils from the repair stage (Supplementary Table 2). Notably, among all transcripts, which were differentially expressed during the inflammatory and the repair stages, the expression of s100a8 and s100a9 was the highest (Supplementary Table 3).

### Colonic eosinophils highly upregulate s100a8 and s100a9 during colonic repair

**Figure 4:**
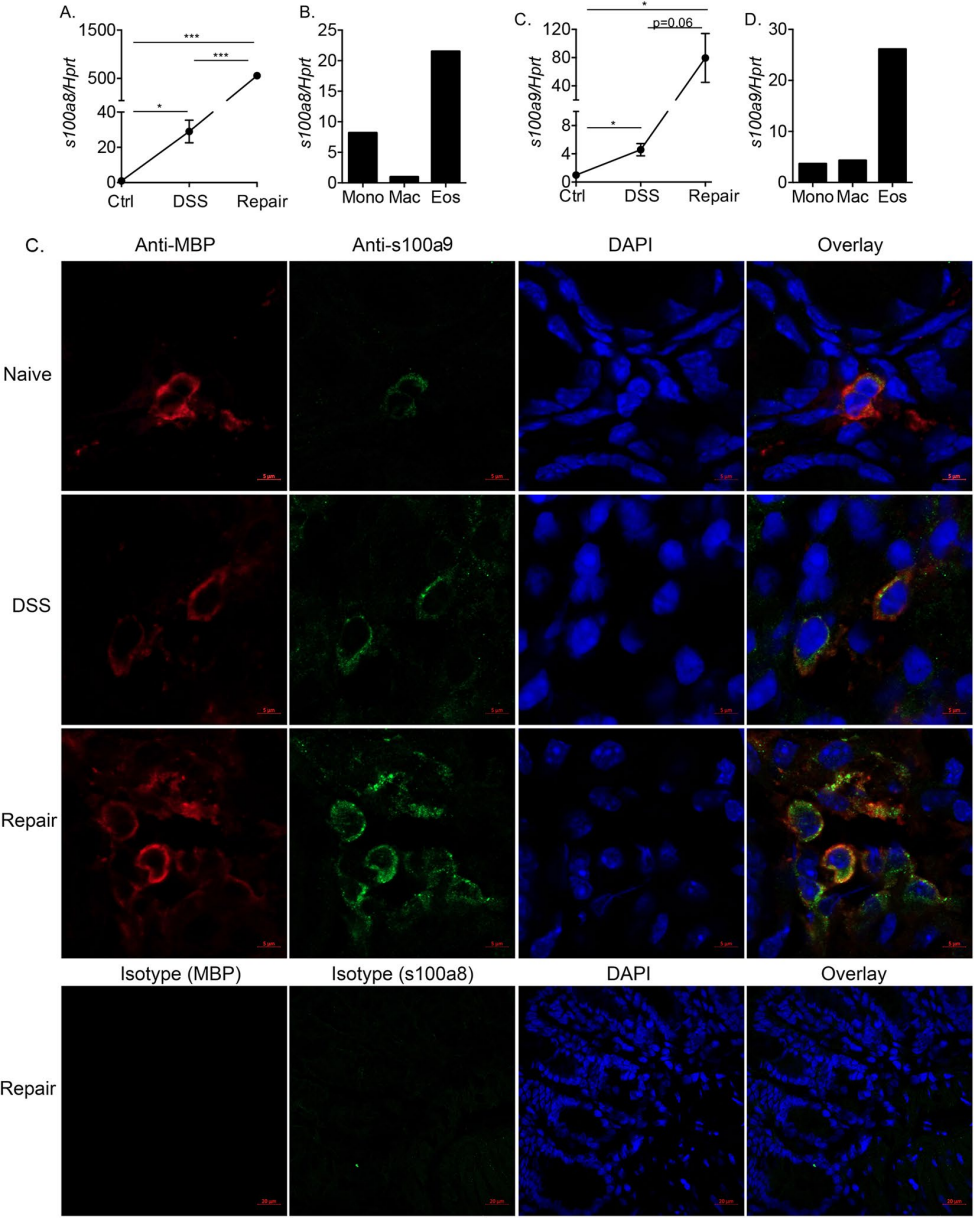
Colonic eosinophils highly upregulate s100a8 and s100a9 during colonic repair. The expression of s100a8 and s100a9 was determined in primary colonic punch biopsies that were obtained from control (Ctrl) mice, and mice during the inflammatory (DSS) and tissue repair stages (**A,C**). The expression of s100a8 and s100a9 in sorted primary colonic eosinophils (Eos), colonic monocytes (Mono) and Macrophages (Mac) was determined by qPCR and normalized to the house keeping gene *Hprt*. Data in (**A,C**) are from n = 3 ***p < 0.001; *p < 0.05, Data in (**B,D**) are from pooled sorted cell. Frozen sections of mouse colons under various conditions were obtained (including baseline, following DSS-treatment and during colonic repair). Thereafter, the slides were stained with anti-s100a9 (Green), anti-MBP (Red) and DAPI (Blue). High-resolution images of single and double stained MBP^+^/s100a9^+^ cells is shown (**D**).

The elevated expression of s100a8 and s100a9 in colonic eosinophils during the inflammatory and repair stages prompted us to further investigate these molecules. Thus, mRNA expression of s100a8 and s100a9 was determined by quantitative PCR analysis in a new cohort of sorted eosinophils. Consistent with our microarray data, the expression of s100a8 and s100a9 was markedly increased following DSS treatment (29.01 ± 11.11- and 4.58 ± 1.51-fold, respectively, Fig. 4A,C). Strikingly, the expression of s100a8 and s100a9 was dramatically increased during the repair stage reaching 555.87 ± 59.35- and 79.55 ± 38.80-fold increase over eosinophils from control mice (Fig. 4A,C).

Our data demonstrated that under baseline conditions eosinophils express low level of s100a8 and s100a9 (Fig. 3E). Thus, we were next interested to assess the relative expression of s100a8 and s100a9 in eosinophils that were taken from the repair stage and additional myeloid cells such as monocytes and macrophages. To this end, colonic monocytes (defined as CD45^+^/Ly6C^+^/CD11b^+^ cells) and macrophages (defined as CD45^+^/CD11b^+^/Ly6C^−^/MHC-II^+^/F480^+^ cells) were sorted and s100a8 and s100a9 expression determined. Indeed, among these cells, the expression of s100a8 and s100a9 was highest in eosinophils (Fig. 4B,D). Notably, although mRNA expression of s100a8 and s100a9 was highly induced in eosinophils, their expression in sorted neutrophils was substantially higher than in eosinophils (5.12- and 8.5-fold increase over eosinophils for s100a8 and s100a9, respectively)^35^.

To definitely demonstrate that eosinophils express and upregulate s100a8, frozen sections of naïve, DSS-treated mice and mice undergoing mucosal healing were obtained and stained with anti-eosinophil MBP as well as anti-s100a9. Under baseline conditions, colonic eosinophils exhibited low but detectable s100a9 expression (Fig. 4E). Following treatment with DSS, and during mucosal repair, increased eosinophilia was observed in the colon and s100a9 expression was markedly increased (Fig. 4E-middle panels).

### Induction of s100a8 and s100a9 is impaired in eosinophil deficient mice

**Figure 5:**
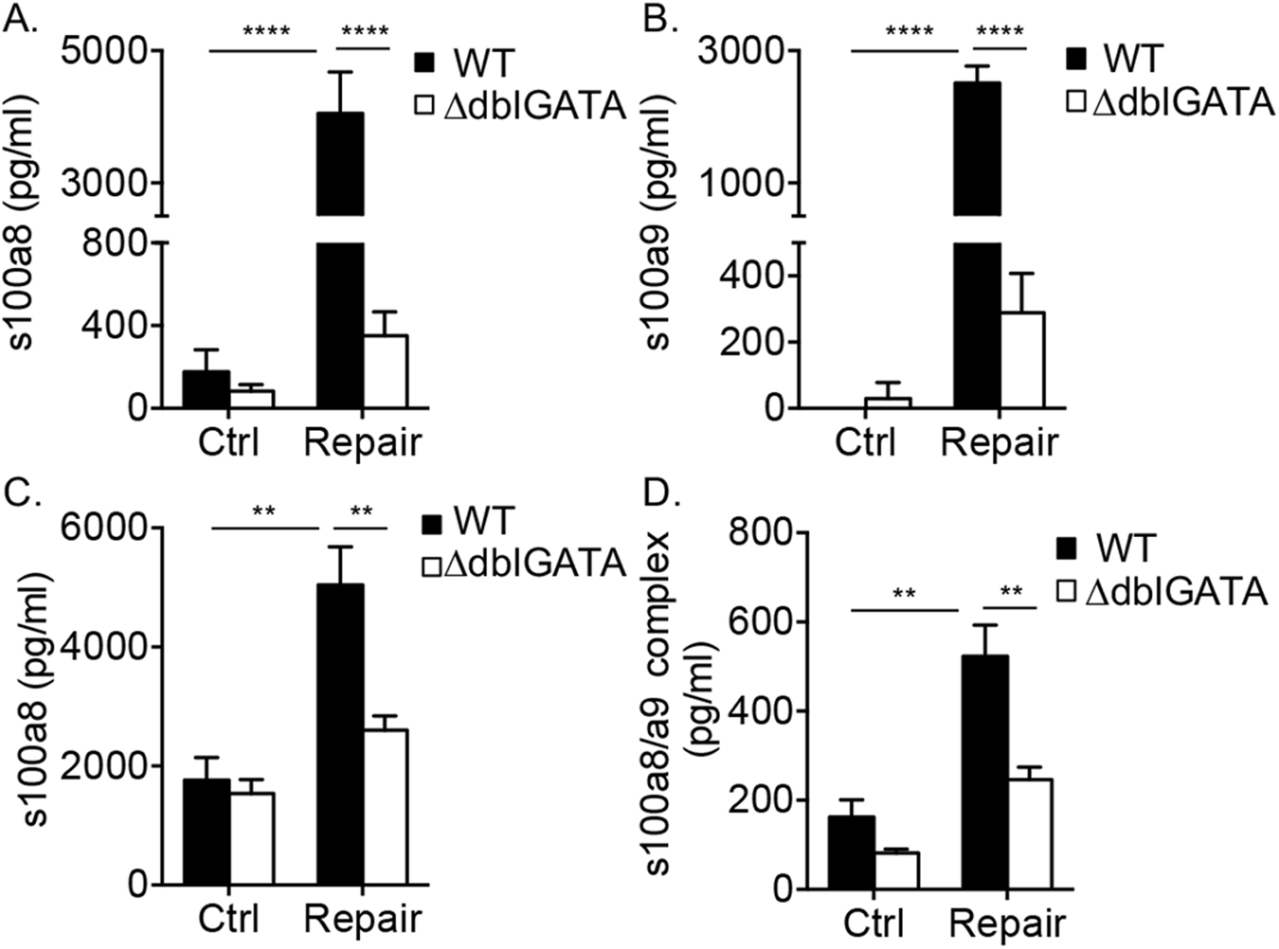
Induction of s100a8 and s100a9 is impaired in eosinophil deficient mice. The expression of monomeric s100a8 and s100a9 (**A–C**) and heterodimeric (**D**) protein forms were determined in the supernatants of colonic punch biopsies (**A,B**) and in the serum (**C,D**) obtained from control (Ctrl) mice, and mice during the inflammatory (DSS) and tissue repair stage. Data are from at least n = 3 experiments, **p < 0.01; ****p < 0.0001.

Next, we were interested to determine whether protein expression of s100a8 and s100a9 during the repair stage was eosinophil-dependent. To this end, colonic “punch” biopsies were obtained from wild type and ∆dblGATA mice and s100a8 and s100a9 expression was assessed. Protein expression of s100a8 and s100a9 was noticeably increased in supernatants obtained from punch biopsies of wild type mice (Fig. 5A,B). Supernatants obtained from ∆dblGATA mice display markedly decreased s100a8 and s100a9 expression (Fig. 5A,B). Furthermore, while wild type mice displayed elevated serum levels of s100a8, ∆dblGATA mice showed nearly no induction (Fig. 5C). Although the commercial ELISA kits that we used are not specific for s100a8 and/or s100a9 homodimers and probably detected mainly the heterodimer with low specificity, each ELISA system clearly showed that the increase in the S100a8 and s100a9 levels was dependent on the presence of eosinophils (Fig. 5A,C).

Given that s100a8 and s100a9 form stable non-covalently associated heterodimers^36^, which are not detected by standard commercial ELISA kits, we were interested to determine whether the serum levels of s100a8/s100a9 heterodimer complex was eosinophil-dependent as well. Certainly, ∆dblGATA mice displayed markedly decreased serum s100a8/s100a9 complexes (Fig. 5D).

### Increased local and systemic expression of s100a8 and s100a9 during mucosal healing is eosinophil-dependent

**Figure 6:**
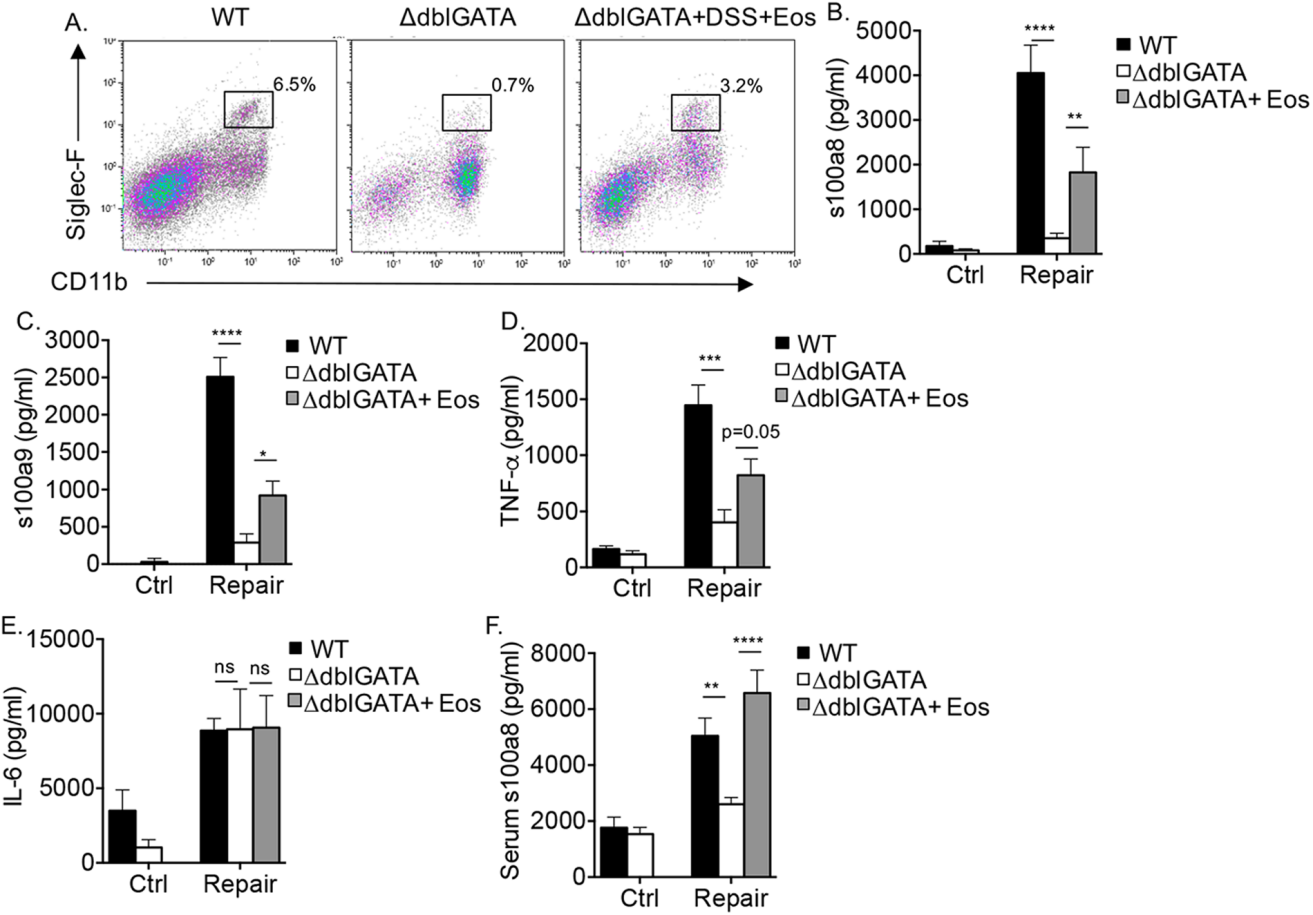
Increased local and systemic expression of s100a8 and s100a9 during mucosal healing is eosinophil-dependent. Eosinophils were adoptively transferred into eosinophil deficient (∆dblGATA) mice during the induction of inflammation by DSS. Gating strategy demonstrating eosinophil levels in wild type (WT) mice, ∆dblGATA mice and ∆dblGATA mice, which received eosinophils during the course of DSS treatment (∆dblGATA + DSS + Eos). Thereafter, the expression of s100a8 (**B,E**) s100a9 (**C**), TNF-α (**D**) and IL-6 (**E**) in colonic punch biopsies (**B–E**) and serum (**F**) obtained from control (Ctrl) mice and mice during the tissue repair stage are shown. Data are from n = 3, ***p < 0.001; **p < 0.01, **p < 0.05.

To definitely determine the contribution of eosinophils to local and systemic s100a8 and s100a9 expression, eosinophils were adoptively transferred into ∆dblGATA mice (Fig. 6A). Reconstitution of eosinophils into the colon of ∆dblGATA mice was capable of significantly increasing the expression of s100a8 and s100a9 in supernatants of colonic “punch” biopsies (Fig. 6B,C). Moreover, eosinophil transfer resulted in elevated levels of TNF-α (Figs 1C and 6D) but not IL-6 (Fig. 6E). Although we were unable to fully reconstitute eosinophil levels to the levels, which were observed in wild type mice (Fig. 6A), systemic expression of s100a8/s100a9 was completely restored (Fig. 6F).

## Discussion

Over the past few years, there is growing interest in understanding the molecular mechanisms underlying mucosal healing^37^. In IBD for example, understanding the molecular basis of mucosal healing could lead to targeted therapy aimed to increase remission which may eventually even impact occurrence of colitis-associated cancer. Eosinophils are resident cells of the GI tract with pleotropic functions. On one hand eosinophils can promote tissue damage and on the other hand promote repair and fibrosis^14^. Although eosinophils emerged as important cells of the innate immune response in mucosal inflammation, their role and their phenotype during intestinal inflammation and repair has been largely overlooked.

Herein, we provide a novel phenotypic analysis of GI eosinophils during acute colonic inflammation and repair. First, we demonstrate that acute colonic inflammation results in marked eosinophilic infiltration into the tissue and that eosinophilia is sustained during later stages of mucosal healing. Thereafter, we show that eosinophils promote intestinal inflammation and therefore eosinophil deficient mice display decreased inflammation and accelerated repair, which is accompanied with decreased levels of TNF-α but not IL-6. Interestingly, using an unbiased global transcriptome analysis approach, we reveal a distinct and potent pro-inflammatory signature for eosinophils during mucosal inflammation that is deactivated during colonic repair. Finally, we establish that eosinophils highly upregulate the expression of s100a8 and s100a9 during colonic healing and that local and systemic expression of s100a8 and s100a9 during colonic healing is largely eosinophil-dependent. Collectively, our data provides unique insight into the genetic signature of eosinophils during colonic inflammation and healing responses and suggest that eosinophils may promote mucosal healing by regulating colonic expression of s100a8 and s100a9.

Our studies demonstrate a potent pro-inflammatory transcriptome signature for eosinophils during the acute phase of DSS-induced colitis. In fact, we show that during the inflammatory stage eosinophils increase the expression of major pro inflammatory cytokines and chemokines including TNF-α, IL-6, IL-1β, CXCL10 and CXCL9. Furthermore, eosinophils upregulate the expression of NFκB-related signaling pathways, which have cardinal roles in this experimental model^38^. This genetic signature is in line with previous findings that demonstrated a pro-inflammatory role for eosinophils in DSS-induced colitis^4–6,9,17^. This inflammatory signature for eosinophils is also consistent with previous studies demonstrating an association between pro-inflammatory cytokines (e.g. IL-6 and TNF-α), s100a8 and s100a9^35,36,39,40^. Interestingly, although eosinophils from the repair stage still displayed increased expression of various pro-inflammatory genes, their overall magnitude (as determined by total inflammatory transcripts numbers and their relative fold increase) was significantly lower than their expression during the acute inflammatory stage and no induction of anti-inflammatory molecules was observed. This result is not trivial since eosinophils are capable of expressing various anti-inflammatory and fibrotic factors including IL-10, VEGF, FGF and TGF-β1^14,41^. In fact, eosinophil-derived TGF-β1 has been associated with the excessive tissue repair and fibrosis, which is observed in patients with asthma and eosinophilic esophagitis^19,42^. Furthermore, eosinophils can promote healing by affecting the vasculature and epithelial cell proliferation^25^. Despite this vast potential to express and secrete growth factors, eosinophils from the tissue repair stage appear to be relatively “de-activated” in comparison with eosinophils from the inflammatory stage. This notion is supported by the finding that the expression of the majority of the pro-inflammatory transcripts, which were induced in inflammatory eosinophils were substantially decreased during the repair phase. This data suggests that factors in the inflammatory/repair milieu (rather than eosinophil-derived factors) act on eosinophils to suppress their activity rather than induce new pro-repair transcripts, which can promote healing. The finding that pro-inflammatory pathways in eosinophils are decreased conforms with the overall notion that “immunologic remission” can occur during mucosal healing. Such immunologic remission has been described by decreased expression of inflammatory cytokine expression (e.g. TNF-α) and increased frequencies of naïve memory T cells^43,44^.

Our microarray analysis revealed that during the inflammatory phase eosinophils increase the expression of various immunoglobulin (Ig)-superfamily receptors (e.g CD300-family members, PIR-B *[Lilrb3*], PILR-α, SIRP-1α^45^) with key immune modulating activities in eosinophils. We have recently shown that CD300f governs eosinophil homing into the gastrointestinal tract and that IL-33 and IL-4 can increase the expression of CD300f in eosinophils and macrophages, respectively^28,46–48^. Furthermore, consistent with increased expression of CD300f in eosinophils from the inflammatory stage, the expression of various CD300-family members including CD300f, CD300a and CD300b was increased in pediatric Crohn’s disease patients associated with the expression of calprotectin^28^. Moreover, we have shown that PIR-B is upregulated in eosinophils and macrophages during settings of intestinal inflammation triggered by IL-13 and innate immune responses, respectively^30,49^. Functionally, PIR-B receptor can potently regulate mucosal inflammatory diseases such as colitis, idiopathic pulmonary fibrosis and asthma^30,50,51^. Therefore, the finding that eosinophils increase the expression of these receptors as a “cluster” during acute inflammatory responses provides further evidence for the importance of these receptors in regulating eosinophil activities^52^.

One of our striking observations was that s100a8 and s100a9 are highly upregulated in eosinophils during inflammation and subsequent repair. Although other cells, especially neutrophils, express s100a8 and s100a9^53^, the expression of s100a8/a9 during colonic inflammation and repair was eosinophil-dependent. Elevation of s100a8 and s100a9 expression in colonic punch biopsies and in the serum, corresponded with the kinetics of increased expression of these molecules in eosinophils. Thus, it is possible that increased local and systemic expression of s100a8 and s1009 is not only eosinophil-dependent but also eosinophil-derived. Alternatively, it is possible that eosinophils regulate the expression of s100a8 s100a9 in other cells by secreting pro-inflammatory factors such as TNF-α that may synergize with IL-17, which has been shown to be elevated following DSS treatment^54,55^. Nonetheless, the finding that the expression of s100a8 and s100a9 is eosinophil dependent is of specific interest. S100a8 and s100a9 are in the focus of rigorous research due to their association with numerous diseases, including acute and chronic inflammatory diseases, autoimmune diseases, cancer, and neurodegenerative diseases^35,36,53^. Interestingly, previous studies have shown a link between the expression of CCL11 (eotaxin-1), a key eosinophil-related chemokine, eosinophils and the expression of s100a8 and s100a9^6^. Eosinophils have been shown to express the receptor for advanced glycation end-products (RAGE), and various RAGE ligands including s100a8 and s100a9^56^. In addition, colonic eosinophils express TLR-4, which is an additional receptor for s100a8/s9^57^. Treatment of eosinophils with IL-5, GM-CSF or lactoferrin, all of which are present in the inflamed and healing mucosa, induced increased expression of s100a8 and s100a9^56,58^. Furthermore, assessment of s100 proteins at the interface of *E. granulosus* larva-host interface revealed that while eosinophils that are distant to the parasite larva express abundant s100a8 and s100a9, the expression of these proteins was largely reduced in proximity to the pathogen^59^. Thus, it appears that the expression of s100a8 and s100a9 is dynamically regulated in eosinophils in response to diverse stimuli especially during mucosal healing. Collectively, this leads to the hypothesis that eosinophil-regulated s100a8 and s100a9 expression participates in various aspects of tissue healing including fibrosis, epithelial cell migration and angiogenesis. While the roles of s100a8/a9 in mucosal healing have been largely overlooked, s100a8 and s100a9 can promote dermal fibroblast proliferation and can facilitate migration of cancer cells via direct and indirect mechanisms^35,53^. Thus, s100a8 and s100a9 may be additional factors contributing to eosinophil-mediated repair processes.

In summary, our study provides a comprehensive phenotypic analysis of eosinophils during colonic inflammation and repair. We highlight eosinophils as important regulators of s100a8 and s100a9 expression during colonic inflammation and mucosal healing, a finding that might have diagnostic and therapeutic implications especially in IBD.

## Methods

### Mice

Wild-type (WT) C57BL/6 mice were originally obtained from Harlan Laboratories (Rehovot, Israel) and grown in-house. CD3-IL-5 transgenic mice (NJ.1638, Il5^Tg^) mice were kindly provided by Dr. Jamie Lee (Mayo Clinic, Scottsdale, AZ). ΔdblGATA mice were kindly provided by Dr. August Avery (Cornell University, Ithaca, NY). All experiments were reviewed and approved by the Animal Care Committee of Tel Aviv University (Number M-13–029, M-13–30), and were performed in accordance with its regulations and guidelines regarding the care and use of animals for experimental procedures. All of the experiments were conducted in the specific pathogen free facilities of the Tel Aviv University. In all experiments, age-, weight-, and sex-matched mice were used.

### Adoptive transfer experiments

Eosinophils were obtained from the spleens of *Il5*^*Tg*^ mice by magnetic bead separation as described^28^. Thereafter, the cells (100 × 10^6^ in 120 μl saline, purity > 95%) were injected intravenously into naïve non-irradiated 6-week old ΔdblGATA mice at day 4 and day 5 of DSS.

### Induction of DSS-colitis and colonic tissue repair

Mice were treated for 5 days with 2.25% dextran sulfate sodium (DSS, MP biomedicals) in their drinking water followed by 14 days of distilled water. For DSS samples, mice were sacrificed at day 5 of DSS treatment; for colonic repair samples, mice were sacrificed at 14 days post DSS cessation.

### Punch Biopsies

The colons were flushed with phosphate-buffered saline and opened along a longitudinal axis; 3 mm^2^ punch biopsies were incubated for 24 h in RPMI supplemented with 10% fetal calf serum and antibiotics. Supernatants were collected and assessed for cytokine expression.

### Enzymatic digestion of gastrointestinal lamina propria cells

Colonic tissue was excised and flushed with 1 ml of calcium- and magnesium-free HBSS (CMF-HBSS). The colon was dissected longitudinally and shaken (250 RPM) in 5 ml CMF-HBSS containing 5% FCS, 2 mM EDTA and 1 mM DTT (Ditiotheritol) for 40 min at 37 °C in order to remove epithelial cells and intraepithelial lymphocytes. Then, the colonic tissue was vortexed and strained through 70 μm grey mesh. The remaining tissue was incubated and shaken (250 RPM) with complete PBS (containing calcium and magnesium) supplemented with 5% FCS, 1 mg/ml collagenase A (Roche, Germany) and 0.1 mg/ml Dnase I (Sigma, Rehovot, IL) for 40 min at 37 °C. The cell suspension was filtered using gauze (70 μm mesh) and suspended in Flow cytometry staining buffer.

### Flow cytometry

Single-cell suspensions of mouse cells were stained using the following antibodies: anti-CD45-APC, anti-CD11b-PerCP-Cy5.5 (obtained from eBioscience, San Diego, CA), anti-Siglec-F-PE (BD Bioscience, San Jose, CA), DAPI (Sigma, St. Louis, MO). Colonic eosinophils were identified as: CD45^+^/CD11b^+^/Siglec-F^+^/SSC^high^.

### Quantitative (q) PCR

RNA samples were subjected to reverse transcription analysis using iScript cDNA synthesis kit (obtained from Bio-Rad, Hercules, CA) according to manufacturer’s instructions. qPCR analysis was performed using the CFX96 system (Bio-Rad laboratories, Hercules, CA) in conjunction with the ready-to-use iQ SYBR Green Supermix (obtained from Bio-Rad, Hercules, CA). Results were normalized to *Hprt* cDNA. The primers that were used in this study were as follows;

*Hprt:* Fwd-GTAATGATCAGTCAACGGGGGAC

Rev-CCAGCAAGCTTGCAACCTTAACCA

*S100a8*: Fwd-CCGTCTTCAAGACATCGTTTGA

Rev-GTAGAGGGCATGGTGATTTCCT

*S100a9*: Fwd-ATACTCTAGGAAGGAAGGACACC

Rev- TCCATGATGTCATTTATGAGGGC

### Enzyme-linked immunosorbent assay (ELISA)

Cytokines were measured by Enzyme-linked immunosorbent assay according to the manufacturer’s instructions (R&D, Minneapolis, MN). Lower detection limits for IL-6, TNF-a, S100a8 and S100a9 were 15.6, 62.5, 62.5 and 62.5 respectively. Detection of s100a8/a9 complex was performed using an in-house ELISA as described^60^. Briefly, wells were coated with the capturing polyclonal antibody anti-s100a8 (4 μg/ml) and polyclonal anti-s100a9 (0.5 μg/ml) antibody coupled to biotin was used as a detection antibody. Purified recombinant murine heterodimer s100a8/a100a9 served as standard.

### Histology

Histological assessment of colonic inflammation and repair was conducted on slides that were obtained from the entire colon and stained with H&E as described^30,61^.

### Immunohistochemistry and immunofluorescence

Anti-MBP was kindly provided by Dr. Jamie Lee (Mayo clinic, Scottsdale, AZ). Secondary Antibody: biotinylated anti-rat (obtained from Vector Laboratory, Burlingame, CA). ABC kit and DAB kit obtained from Vector Laboratory. Counterstain performed with nuclear fast red (obtained from Sigma, St. Louis, MO). For immunofluorescence studies, frozen colon sections of naïve, DSS-treated mice and mice undergoing mucosal repair were obtained as described (ref). Thereafter, the tissue was stained with anti- MBP (1:500), anti-s100a9 (anti-MRP14, 1:500) or isotype controls, followed by goat anti-rat AlexaFluor 647 (1:350, Jackson ImmunoResearch, West Grove, PA) and goat anti-rabbit DyLight 488 (1:400, Jackson ImmunoResearch, West Grove, PA), respectively. Images were captured using an Olympus AX70 fluorescent microscope (Center Valley, PA, USA) equipped with a DP72 camera.

### Affymetrix cDNA microarray

For microarray experiments, eosinophils were sorted from 5–10 mice per group immediately following enzymatic digestion and RNA was extracted freshly after cell isolation. In all of these experiments, 50,000 cells per sample were collected at a purity of >95%. RNA was extracted using RNAqueous-Micro kit (obtained from Invitrogen, Carlsbad, CA) according to manufacturer’s instruction. RNA quality was assessed using a bioanalyzer (Agilent 2100) and achieved an RNA quality number (RIN) of at least 7. RNA was then amplified, fragmented and labeled using the Ovation Pico WTA System V2 and the Encore Biotin Module (obtained from Nugen, San Carlos, CA). Mouse Affymetrix (Santa Clara, CA) microarrays (2.1 ST GeneChip) were performed and analyzed using established protocols of the Tel-Aviv University Bioinformatics Unit and according to the manufacturer’s instructions. Data were analyzed using Partek (Partek, St. Louis, MO) and Genespring GX (Agilent Technologies, Santa Clara, CA).

### Statistical analysis

Data were analyzed by analysis of variance followed by Tukey *post hoc* test or Student’s *t*-test using GraphPad Prism 5 (obtained from GraphPad, San Diego, CA). Data are presented as mean ± s.e.m, and values of *P* < 0.05 were considered statistically significant.

## Electronic supplementary material

Supplementary Information
