# Author Correction: Transcriptome profiling of mouse colonic eosinophils reveals a key role for eosinophils in the induction of s100a8 and s100a9 in mucosal healing

**DOI:** 10.1038/s41598-018-26377-6

**Published:** 2018-05-23

**Authors:** Hadar Reichman, Italy Moshkovits, Michal Itan, Metsada Pasmanik-Chor, Thomas Vogl, Johannes Roth, Ariel Munitz

**Affiliations:** 1 Department of Clinical Microbiology and Immunology, The Sackler School of Medicine, Tel-Aviv University, Ramat Aviv, 69978 Israel; 2 Bioinformatics Unit, George S. Wise Faculty of Life Sciences, Tel Aviv University, Tel Aviv, 64239 Israel; 3 Institute of Immunology, University of Münster, Münster, Germany

Correction to: *Scientific Reports* 10.1038/s41598-017-07738-z, published online 02 August 2017

The original version of this Article contained errors in the spelling of the authors Hadar Reichman, Italy Moshkovits, Michal Itan, Metsada Pasmanik- Chor, Thomas Vogl, Johannes Roth & Ariel Munitz which were incorrectly given as Reichman Hadar, Moshkovits Itay, Itan Michal, Pasmanik-Chor Metsada, Vogl Thomas, Roth Johannes & Munitz Ariel.

In addition, in the original version of this Article, Figures 1 and 2 were inadvertently published as Figures 2 and 1. The Figure legends were correct from the time of publication.

These errors have now been corrected in the PDF and HTML versions of the Article and the Supplementary Information file that accompanies the Article.

