# Supplementary material for: Transcriptome profiling of mouse colonic eosinophils reveals a key role for eosinophils in the induction of s100a8 and s100a9 in mucosal healing
Source: Sci Rep. 2017 Aug 2;7:7117. doi: 10.1038/s41598-017-07738-z (PMC5540981; doi:10.1038/s41598-017-07738-z)
Supplement: Supplementary file 1 — Supplementary Information [file 41598_2017_7738_MOESM1_ESM.pdf]

Supplementary Data Online

Transcriptome profiling of mouse colonic eosinophils reveals a key role for eosinophils in the induction of s100a8 and s100a9 in mucosal healing.

Hadar Reichman [1], Itay Moshkovits [1], Michal Itan[1], Metsada Pasmanik-Chor[2], Thomas Vogl [3], Johannes Roth[3], Ariel Munitz[1,*]

[1] Department of Clinical Microbiology and Immunology, The Sackler School of Medicine, Tel-Aviv University, Ramat Aviv 69978, Israel.

[2] Bioinformatics Unit, George S. Wise Faculty of Life Sciences, Tel Aviv University, Tel Aviv, 64239, Israel

[3] Institute of Immunology, University of Münster, Münster, Germany

Running Title: mRNA profiling of gastrointestinal eosinophils

*Corresponding author: Ariel Munitz, PhD, Department of Clinical Microbiology and Immunology, The Sackler School of Medicine, Tel-Aviv University, Ramat Aviv 69978, Israel. Tel. (Office): +972-3-640-7636, Fax: +972-3-640-9160, e-mail: arielm@post.tau.ac.il.

Table S1. Genes that are uniquely up/downregulated in eosinophils following DSS treatment

Gene ID	Fold Change
2010002N04Rik	2.114535
2210415F13Rik	-2.73094
AB124611	2.598496
Acta2	-2.86843
Adh1	-4.88427
Adrb2	2.06037
Ahr	2.116352
Alox5ap	3.20226
Arhgap15	2.385151
Arhgdib	3.641349
Arih2	2.353609
Art2a-ps	2.190812
Atp1b1	-2.38681
BC013712	2.87252
Bcl2a1a	2.793273
Bcl2a1b	2.441244
Bcl2a1c	2.261608
Bcl2a1d	2.491155
Birc3	2.068773
Btg1	2.203661
C5ar1	2.076396
Camk1	2.133913
Car2	-2.89431
Car4	-3.04983
Ccl3	2.602995
Ccl4	2.343079
Ccl6	2.332342
Ccr1	2.405439
Ccr3	3.774776
Ccrl2	2.669071
Cd274	3.644429
Cd300a	2.209071
Cd300ld	2.069237
Cd52	3.413551
Cd53	2.616041
Cd84	2.278048
Cd9	2.065873

Cd97	2.571372
Ckmt1	-3.75917
Cks2	2.331461
Coro1a	2.541166
Cotl1	3.035732
Creg1	2.482882
Csf2rb	3.63979
Ctsd	2.150735
Ctsz	2.072115
Cxcr4	2.216787
Cyba	2.908619
Cybb	4.201919
Cyld	2.149647
Cytip	2.202597
Dapp1	2.747108
Dennd3	2.039229
Dennd4a	2.840187
Dennd5a	2.172381
Ehd1	2.16642
Elovl5	2.007339
Emr1	2.749504
Entpd1	2.815916
F5	2.581218
Fam65b	2.221272
Fas	2.647638
Fcer1g	3.776913
Fcgr3	3.267153
Fem1c	2.166504
Fxyd5	2.665104
Fyb	3.170652
G0s2	2.05425
Gdap10	3.223841
Gm5150	2.250391
Gmfg	2.756347
Gpr132	2.251465
Gpr65	2.782211
Grina	2.407612
Gsr	2.258602
Guca2a	-3.34513
H2-Aa	2.391259
H2-D1	2.15917

H2-Q6	2.752537
H2-Q7	2.08012
Hck	2.324554
Hcls1	2.297287
Hdc	4.22893
Icam1	2.322812
Ifnar2	2.457283
Igfbp7	-2.21895
Il1r2	2.059174
Il2rg	2.559991
Irak3	2.365692
Irg1	3.225836
Itga4	2.755472
Itgal	2.127228
Itgam	3.104096
Klk1	-4.10211
Klk1b21	-2.05891
Laptm5	2.913389
Lcp1	2.709654
Lcp2	3.305294
Lilrb3	2.210415
Litaf	2.333824
Lpcat2	2.552466
Lst1	2.880612
Lyn	2.335944
Man2b1	2.329557
Mapkapk2	2.979047
Mgst3	-2.157
Muc13	-2.09953
Muc3	-4.54266
Myh11	-2.09352
Myo1f	2.357083
Nampt	2.745428
Ncf1	4.05721
Ncf2	2.443366
Ncf4	2.390266
Nfkb1	2.09586
Nfkbia	2.024548
Nfkbie	2.0962
Ninj1	3.094667
Oit1	-3.33384

Phgr1	-2.11442
Pik3ap1	2.611873
Pilra	2.752529
Pilrb1	2.764565
Pilrb2	2.30407
Pim1	2.553747
Pira1	2.866859
Pira11	2.580348
Pla2g7	2.379898
Plek	3.312831
Plxnc1	2.281245
Pot1b	2.114319
Psmb9	2.154104
Pstpip2	2.892921
Ptafr	3.024613
Ptprc	3.195784
Rab44	2.46207
Rab8b	2.200054
Rassf4	2.391502
Rel	2.216503
Retnlg	3.024982
Rhog	2.429441
Rilpl2	2.686094
Rnf19b	2.598385
Samhd1	3.675036
Samsn1	2.365456
Serpine1	2.845712
Sfpi1	2.720064
Sh3bgrl3	2.478338
Sh3kbp1	2.144873
Sirpa	2.508805
Sla	2.453402
Slc7a11	2.207467
Snap23	2.37133
Sorl1	2.0915
Sparc	-3.11886
Sparcl1	-2.47993
Spink4	-2.95548
Sprr2a1	-2.29804
Stat1	2.432221
Sykb	2.411535

Syne1	2.291328
Tgfbi	2.552116
Tgm2	2.034245
Tmem123	2.057721
Tmem71	2.468822
Tnf	2.520313
Tnfaip3	2.316878
Tpm1	-2.08361
Treml4	2.037618
Tspan1	-2.4778
Tyrobp	3.008356
Zeb2	2.022895

Table S2. Genes that are uniquely up/downregulated in eosinophils in the repair stage

Gene ID	Fold Change
C3	2.603751
Expi	3.458157
Hp	2.221408
Lcn2	5.628583
Lrg1	2.182371
Ngp	2.02339
Retnlb	3.220213
Rgs1	-2.11222
Stfa2l1	2.901982
1600029D21Rik	3.842435
1810065E05Rik	2.210057
2210407C18Rik	2.118108
AW112010	2.574002

Table S3. Genes that are up/downregulated in eosinophils following DSS and during the repair stage

	Fold Change	
**Gene ID**	**DSS**	**Repair**
Cd14	3.572129	3.475782
Clca3	-4.70141	-3.53164
Clec4e	4.016285	2.624751
Cxcl10	3.368698	2.113798
Cxcl9	4.371846	3.374673
Gbp2	3.286584	2.235264
Gm11428	5.28443	5.89317
Ifitm1	3.372672	2.004154
Igsf6	4.80966	2.080996
Il1a	3.451913	2.263577
Il1b	3.042111	2.396904
Il6	2.660003	2.242947
Ptgs2	3.138158	2.261632
Reg3b	3.333324	3.97256
Reg3g	5.790898	7.923525
Rgs5	-2.74151	-2.01197
S100a8	8.214421	45.32977
S100a9	11.60171	34.28278
Saa3	2.773518	7.781145
Slfn2	4.155291	2.13804
Slpi	2.314184	2.144377
Sycn	-7.71414	-4.19286
Tgm3	-3.08712	-2.45984
